# DFT Study of Molecular and Electronic Structure of Ca(II) and Zn(II) Complexes with Porphyrazine and tetrakis(1,2,5-thiadiazole)porphyrazine

**DOI:** 10.3390/ijms21082923

**Published:** 2020-04-22

**Authors:** Arseniy A. Otlyotov, Igor V. Ryzhov, Ilya A. Kuzmin, Yuriy A. Zhabanov, Maxim S. Mikhailov, Pavel A. Stuzhin

**Affiliations:** Ivanovo State University of Chemistry and Technology, Research Institute of Chemistry of Macroheterocyclic Compounds, 153000 Ivanovo, Russia; otlyotov@isuct.ru (A.A.O.); ryzhoff.ihor@yandex.ru (I.V.R.); wonderful_37@list.ru (I.A.K.); mihailov_maxim_s@mail.ru (M.S.M.); stuzhin@isuct.ru (P.A.S.)

**Keywords:** porphyrazine, 1,2,5-thiadiazole annulated, DFT study, molecular and electronic structure

## Abstract

Electronic and geometric structures of Ca(II) and Zn(II) complexes with porphyrazine (Pz) and tetrakis(1,2,5-thiadiazole)porphyrazine (TTDPz) were investigated by density functional theory (DFT) calculations and compared. The perimeter of the coordination cavity was found to be practically independent on the nature of a metal and a ligand. According to the results of the natural bond orbital (NBO) analysis and quantum theory of atoms in molecules (QTAIM) calculations, Ca–N bonds possess larger ionic contributions as compared to Zn–N. The model electronic absorption spectra obtained with the use of time-dependent density functional theory (TDDFT) calculations indicate a strong bathochromic shift (~70 nm) of the Q-band with a change of Pz ligand by TTDPz for both Ca and Zn complexes. Additionally, CaTTDPz was synthesized and its electronic absorption spectrum was recorded in pyridine and acetone.

## 1. Introduction

Porphyrins, phthalocyanines and their analogues have found a number of applications, particularly, due to their intense absorption in the visible region [1,2,3,4]. Since the optical properties are governed by the electronic structure of the macrocycle, thorough theoretical studies by quantum-chemical methods are usually performed to explain the observed features of the absorption spectra [5,6,7,8,9,10,11,12,13] and open the possibilities of their in-silico design in the case of compounds, for which the experimental data are absent. Such investigations in the case of the complexes with transition metals are often non-trivial due to the necessity to account for the multireference character of the wavefunction. However, in the case of the closed-shell species, density functional theory (DFT) can be directly applied to obtain the qualitative and quantitative information about the ground-state properties. Therefore, a reasonable first step in the comparative studies of the influences of a transition metal and a ligand on the chemical bonding and spectral properties is to consider the relatively simple borderline *d*^0^ and *d*^10^ configurations (Ca and Zn, respectively) in order to eliminate the multireference effects.

**Figure 1:**
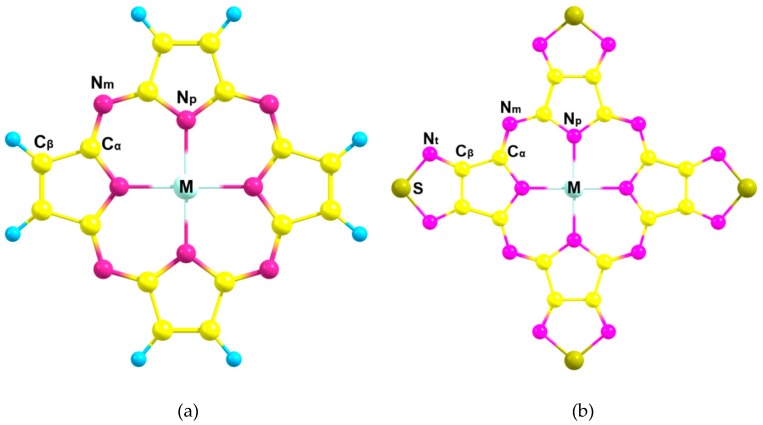
Molecular models of M-porphyzarine (MPz) (**a**) and M-tetrakis(1,2,5-thiadiazole)porphyzarine (MTTDPz) (**b**) complexes with atom labeling (M = Ca, Zn).

While porphyrins and phthalocyanines have been widely investigated, the information on their porphyrazine (Pz) analogues is still incomplete. Moreover, in recent years, much attention has been paid to 1,2,5-thiadiazole-fused porphyrazines possessing especially strongly electron-deficient macrocycle, and capable of forming layers with strong intermolecular interactions. As a result, tetrakis(1,2,5-thiadiazole)porphyrazine (TTDPz) and its metal complexes are actively studied for application in organic electronics, such as n-type semiconductors [14,15,16,17,18]. Therefore, their theoretical study is quite important to reveal the influence of 1,2,5-thiadiazole rings on the peculiarities of the electronic properties of the porphyrazine macrocycle in the metal complexes (Figure 1) with different contributions of σ- and π-bonding effects in the formation of M-N_p_ bonds [2,3,7,17,19,20].

Earlier in our laboratory, the magnesium (II) complexes with tetrakis(1,2,5-chalcogenadiazole) MgTXDPz (X = O, S, Se, Te) were investigated by DFT calculations in order to examine the influence of a chalcogen atom on their geometry and electronic structure [21]. The theoretical studies of the molecular structures and electronic spectra of the porphyrazine complexes with the alkaline-earth metals Be and Mg are described in [13], and for the porphyrazine complexes with alkali metals in [22]. The present contribution aims to determine the nature of the chemical bonding and influence of the metal atom (Ca [*d*^0^] and Zn [*d*^10^]) and the ligand (Pz and TTDPz) on the electronic absorption spectrum. It should be mentioned that the electronic spectrum of ZnPz complex has already been thoroughly interpreted in [7,11]. We recalculated it using a different theoretical approximation only for comparison purposes. Besides, in order to complement the comparison, a CaTTDPz complex was synthesized for the first time and its electronic spectrum was measured.

## 2. Results and Discussion

### 2.1. Chemical Bonding in MPz and MTTDPz

The closed-shell MPz and MTTDPz complexes with Ca and Zn can be treated using single-reference methods. Therefore, DFT was chosen for all calculations. The equilibrium structures of the complexes ZnPz and ZnTTDPz were determined to possess the planar structures of D_4h_ symmetry, while the complexes with Ca(II) exhibit significant doming distortion, and their structures belong to the *C*_4v_ point group. The force-field calculations yielded no imaginary frequencies, indicating that the optimized configurations correspond to the minima on the potential energy hypersurfaces. The calculated molecular parameters are presented in Table 1.

The results of the natural bond orbital (NBO) analysis of the electron density distribution demonstrate the different nature of chemical bonding in the MPz and MTTDPz complexes. First, we find a decrease of the ionic component of M–N bond in the case of the d^10^ shell of Zn(II), as compared to the Ca(II) complex with an unoccupied d^0^ shell. This can be rationalized not only in terms of the Wiberg bond index Q(M-N), which increases from Ca–N to Zn–N, but also by the comparison of the energies of donor–acceptor interactions (∑ E(d-a)) between lone pairs on the nitrogen atoms and 4s-, 3d- and 4p- orbitals of the metal atoms. Another confirmation stems from the values of the delocalization indices calculated in the framework of the quantum theory of atoms in molecules (QTAIM) analysis being close to the values of Q(M-N).

**Figure 2:**
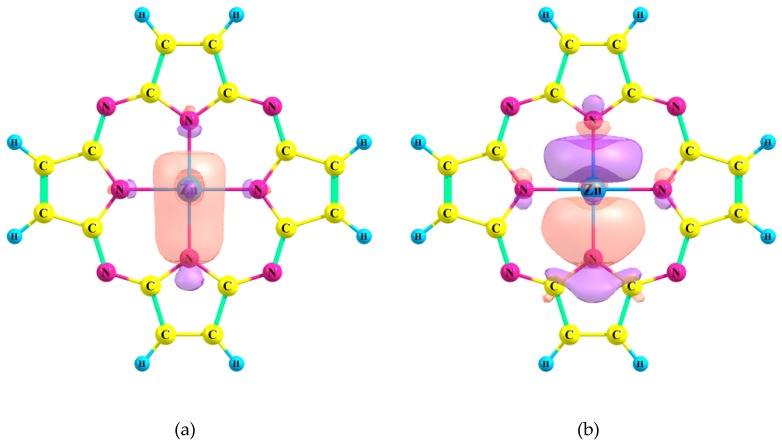
Schemes of the dominant donor-acceptor interactions between Zn and Pz ligand: (**a**) the result of the orbital interaction of the type LP(N) → 4*s*(Zn) (E^(2)^ = 54.0 kcal mol^−1^); (**b**) the result of the orbital interaction of the type LP(N) → 4*p*(Zn) (E^(2)^ = 61.9 kcal mol^−1^). Only one of the four corresponding interactions is demonstrated.

**Figure 3:**
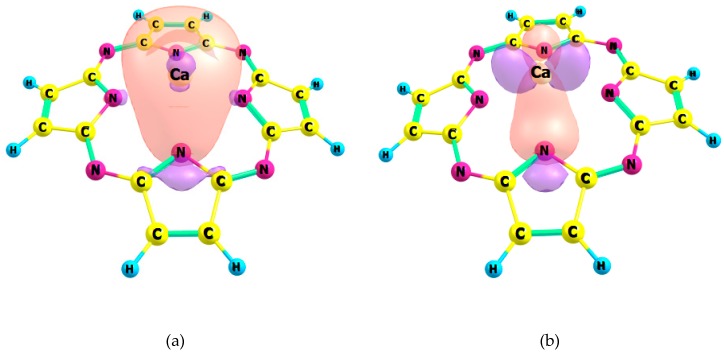

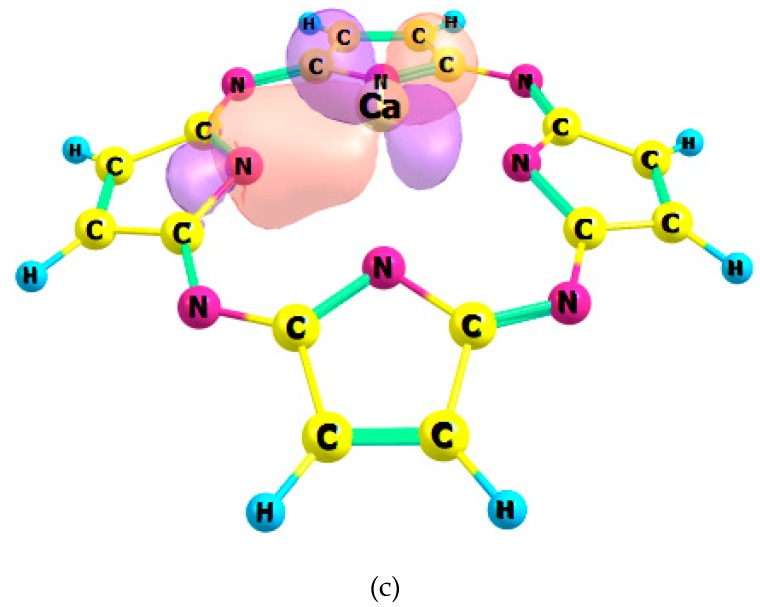
Schemes of the dominant donor-acceptor interactions between Ca and Pz ligand. The results of the: (**a**) orbital interaction of the type LP(N) → 4s(Ca) (E^(2)^ = 11.0 kcal mol^−1^); (**b**) orbital interaction of the type LP(N) → 3d_x_^2^_−y_^2^(Ca) (E^(2)^ = 3.5 kcal mol^−1^); (**c**) orbital interaction of the type LP(N) → 3d_yz_(Ca) (E^(2)^ = 3.9 kcal mol^−1^).

The complexes of the Pz and TTDPz ligands with Zn(II) are stabilized by strong interactions of these types: LP(N) → 4s(Zn) and LP(N) → 4p(Zn) (Figure 2). In the case of the Ca(II) complexes, only much weaker interactions LP(N) → 4s(Ca), LP(N) → 3d_x_^2^_−y_^2^(Ca) and LP(N) → 3d_yz_(Ca) were found within the NBO scheme (Figure 3).

Interestingly, while the Zn(II) complexes are stable even in concentrated H_2_SO_4_ in ambient conditions [23], the Ca(II) complex with TTDPz macrocycle, first prepared in the present work, undergoes easy demetalation upon treatment with hot acetic acid, and forms ZnTTDPz upon heating with the Zn(II) acetate in pyridine. This experimental observation is confirmed theoretically (within the rigid rotor–harmonic oscillator (RRHO) approximation from the B3LYP/pcseg-2 geometries and the harmonic frequencies) by the large negative value of the Gibbs free energy (∆_r_*G*⁰(298.15) = −678 kJ mol^−1^) of the reaction: CaTTDPz + Zn^2+^ → Ca^2+^ + ZnTTDPz. The analogous value for the reaction CaPz + Zn^2+^ → Ca^2+^ + ZnPz is ∆_r_*G*⁰(298.15) = −695 kJ mol^−1^.

In the framework of the QTAIM theory, the existence of a chemical bond indicates the presence of a bond critical point (BCP) between the corresponding atoms. The nature of the chemical bond can be determined by the value of the electron density, laplacian ∇^2^ρ. A positive value of the electron density laplacian ∇^2^ρ indicates ionic interaction. However, the values of M-N_p_ bond orders, as well as the corresponding delocalization indices δ(M|N_p_) representing the magnitudes of the electron exchange between the basins of the corresponding atoms, allow to argue that these bonds, along with an ionic component (Table 2), possess a noticeable covalent component.

The annelated thiadiazole ring in the TTDPz complex also influences the geometry of the coordination cavity. The electron density is shifted towards electron-withdrawing nitrogen atoms in the thiadiazole moieties. It in turn leads through the inductive effect to a charge transfer in the row N_t_ ← C_β_ ← C_α_. The weakening of the N– C_α_ bonds results in an increase of the C_α_–N–C_α_ angle and the elongation of M–N distance in the MTTDPz complexes as compared to their MPz analogues.

**Figure 4:**
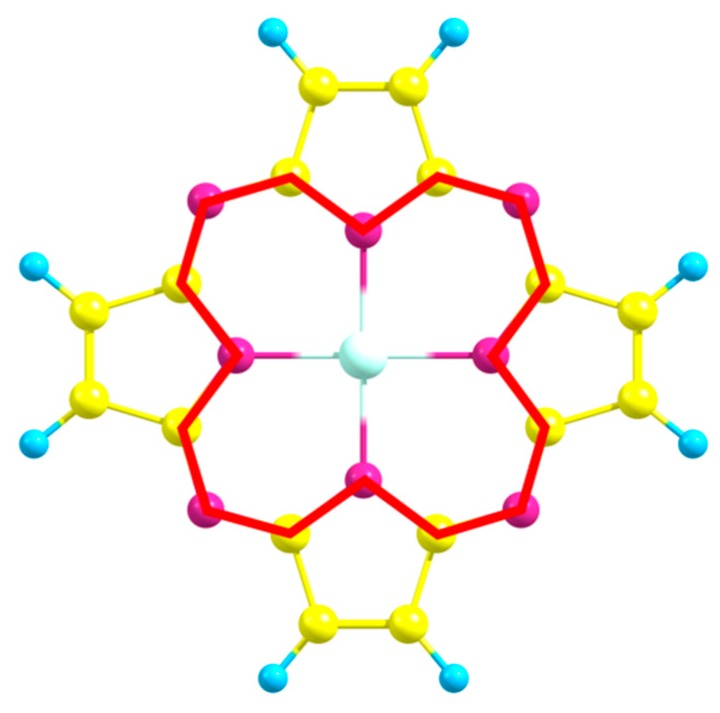
Internal macrocycle perimeter.

As it was previously found for the complexes of La and Lu with hemihexaphyrazine [24], the perimeters of the internal 16-membered macrocycle of all the studied structures (Figure 4) do practically not depend on the nature of a metal atom, and are equal to 21.55(2) Å.

### 2.2. Molecular Orbitals

**Figure 5:**
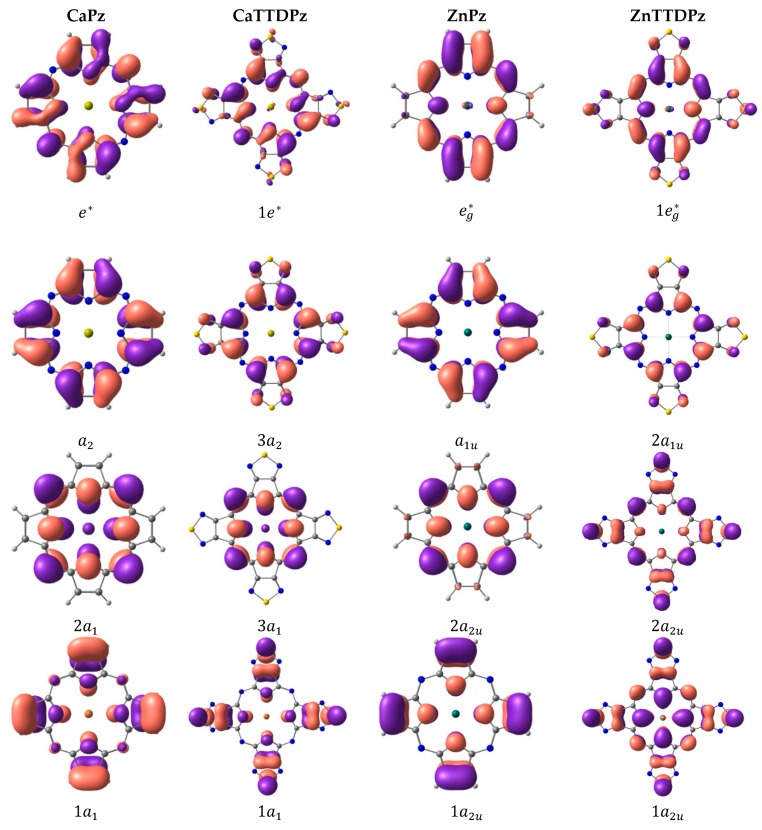
Influence of the metal (Ca/Zn) and ligand (Pz/TTDPz) on the molecular orbitals of MPz and MTTDPz complexes.

The symmetry of the frontier molecular orbitals is similar in the ZnPz and ZnTTDPz complexes, and is also typical for porphyrzines: the highest occupied molecular orbital (HOMO) is an a_1u_ orbital and the lowest unoccupied molecular orbitals (LUMOs) are doubly-degenerated e_g_* orbitals (Figure 5). The LUMOs are localized on the porphyrazine macrocycle. The situation is similar for the calcium complexes but different in the symmetry of orbitals (for example, the HOMO is an a_2_ orbital and the LUMOs are doubly-degenerated e*) due to another symmetry point group.

The nodes of the HOMO are located on the carbon atoms in the case of Pz complexes and additionally on the N_t_ atoms for TTDPz macrocycles. The separation of the HOMO from the other π-MOs is less pronounced in the case of Pz complexes as compared to their thiadiazole-annelated analogues.

**Figure 6:**
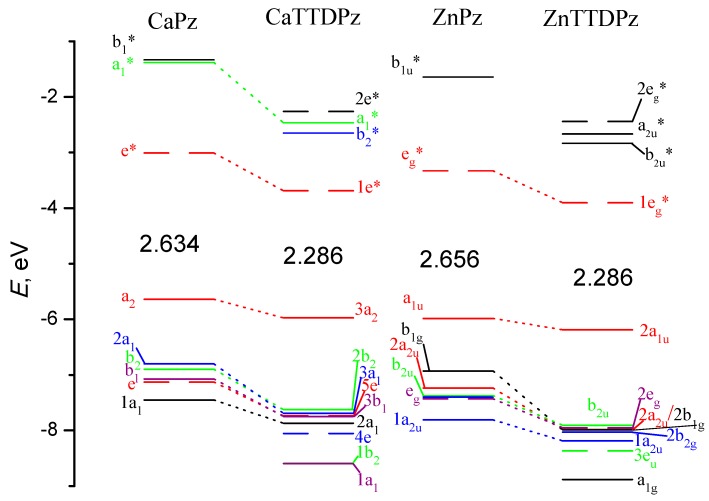
Molecular orbital (MO) level diagram for MPz and MTTDPz complexes (M = Ca, Zn). The values of highest occupied molecular orbital-lowest unoccupied molecular orbital (HOMO-LUMO) gaps are given in eV.

The HOMO-1 MO in CaPz, the HOMO-2 in CaTTDPz and ZnPz, and the HOMO-4 in ZnTTDPz are Gouterman type orbitals [25,26] predominantly localized on the nitrogen atoms of the macrocycles, except for ZnTTDPz. They can be connected with a significant decrease of the energy of this orbital in the case of ZnTTPz as compared to the other molecules (Figure 6).

### 2.3. Electonic Absorption Spectra

**Figure 7:**
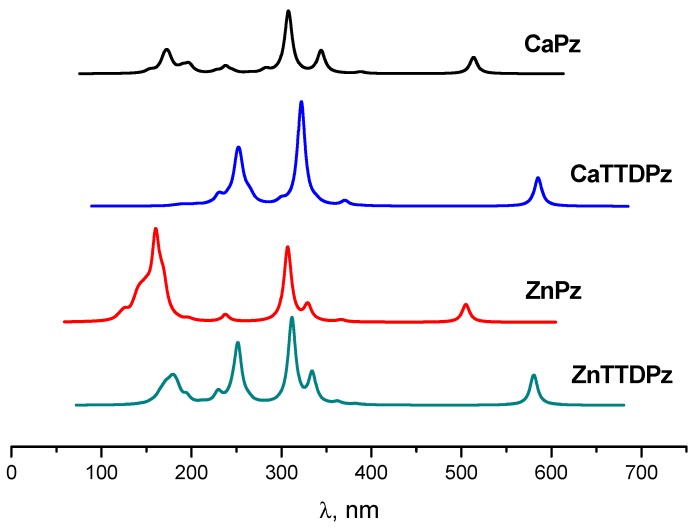
Calculated TDDFT electronic absorption spectra for MPz and MTTDPz complexes.

The comparison of the calculated spectra demonstrates a strong influence of the ligand. For both Ca and Zn complexes, a strong bathochromic shift (~70 nm) of the Q-band occurs with a change of Pz ligand by TTDPz (Figure 7). The calculated oscillator strengths (*f*) for the lowest-allowed excited states along with their composition (in terms of one-electron transitions) are given in Table 3.

The long-wave absorption maxima (Q band) in the spectra of MPz and MTTDPz can be assigned to the almost pure Goutermantype [25,26] transition $\;a_{2}\to e_{g}^{*}$ for Ca complexes and $a_{1u}\to e_{g}^{*}$ for Zn complexes. The electronic transitions to the higher excited states (the Soret near-UV region of 300–420 nm) possess larger oscillator strengths and are predominantly composed of transitions from the filled $a_{1}$ (Ca complexes) $a_{2u}$ (Zn complexes) type MOs to the LUMOs.

## 3. Computational Methods

The DFT-based investigation of MPz and MTTDPz included geometry optimizations and computations of the harmonic vibrations followed by TDDFT calculations of the electronic absorption spectrum. The number of the calculated excited states was 30. The calculations were performed using B3LYP functional and pcseg-2 basis set [28] taken from the EMSL BSE library [29,30]. The Firefly QC [31] package, which is partially-based on the GAMESS(US) [32] source code was used in all the calculations. Optimized Cartesian coordinates of MPz and MTTDPz are available from Supplementary materials.

The QTAIM (quantum theory of atoms in molecules) analysis [33] was performed using the AIMAll [34] software package. Topological parameters of *ρ*(r) in bond critical points and charges on atoms are collected in Supplementary materials.

The molecular models and orbitals demonstrated in the paper were visualized by means of the Chemcraft program [35].

## 4. Experimental

### Synthesis of CaTTDPz

Calcium metal (0.35 g, 8.5 mmol) was refluxed in 50 mL of butanol in a round-bottom flask for 12 h affording the suspension of Ca(II) butoxide. Further 3,4-dicyano-1,2,5-thiadiazole (1.15 g, 8.5 mmol) was added and the reaction mass was refluxed with vigorous stirring for 8 h. At the end of the synthesis, the reaction mixture was poured into a Petri dish and left until the butanol was completely evaporated. Further, the solid mass was washed with CH_2_Cl_2_ to remove the unreacted dinitrile and low molecular weight reaction intermediates. After drying, the resulting product was poured into a 25% aqueous solution of acetic acid, and at room temperature with continuous stirring it was held for 1 h to dissolve the calcium butoxide. The solid precipitate was filtered and washed repeatedly with water and then with acetone and dried to constant weight. The mass of the obtained product is 0.7 g (yield 45%). Electronic absorption spectra of CaTTDPz in pyridine and acetone are given in Supplementary materials.

## 5. Conclusions

The influence of the nature of the metal (either Ca or Zn) and the ligand (either porphyrazine or thiadiazole-annelated porphyrazine) on the geometry and electronic structure of the macroheterocyclic complex was studied with the use of DFT calculations at the B3LYP/pcseg-2 level. The nature of the chemical bonding is quite different in the case of Zn complexes as compared to the Ca analogues. Overall, all the complexes have a substantial ionic contribution to the M-N_p_ bonding, but a much larger covalent contribution appears in ZnPz and ZnTTDPz due to the donor-acceptor interactions of the type LP(N) → 4s(Zn) and LP(N) → 4p(Zn). The perimeter of the coordination cavity was found to be practically independent on the nature of a metal and a ligand.

The change of Pz ligand by TTDPz causes a strong bathochromic shift (~70 nm) of the Q-band for both Ca and Zn complexes. As it usually occurs to porphyrazine metal complexes, the Q-band can be assigned to the almost pure Gouterman type transition.

While the complexes of porphyrazine with Mg(II) are easily accessible and well-studied, the Ca(II) complexes are not known. In this work, we prepared the CaTTDPz complex for the first time and demonstrated that it possesses high lability. This is explained theoretically by the more ionic nature of the N_p_-Ca bonds as compared to the N_p_-Zn bonds. Unlike the Ca(II) complex, the Zn(II) complex cannot be prepared directly by the template cyclotetramerization of the dinitrile, but instead can be obtained readily from the Ca(II) complex.

## Figures and Tables

**Table 1:** Molecular parameters ^1^ of M-porphyzarine (MPz) and M-tetrakis(1,2,5-thiadiazole)porphyzarine (TTDPz) complexes optimized at B3LYP/pcseg-2 level.

	CaPz	CaTTDPz	ZnPz	ZnTTDPz
M-N_p_	2.276	2.299	1.979	2.025
M-X ^2^	1.079	1.020		
N_p_-C_α_	1.364	1.373	1.363	1.375
C_α_-C_β_	1.458	1.462	1.457	1.458
C_α_-N_m_	1.333	1.322	1.331	1.317
C_β_-C_β_	1.354	1.424	1.457	1.421
C_β_-N_t_		1.316		1.316
N_t_-S		1.645		1.644
(N_p_…N_p_)_opp_	4.008	4.120	3.958	4.049
(N_p_…N_p_)_adj_	2.834	2.913	2.799	2.863
∠ (N_p_–M–N_p_)	123.4	127.3	180.0	180.0
∠ (N_p_–C_α_–N_m_)	127.6	128.1	127.2	128.0
∠ (C_α_–N_m_–C_α_)	124.6	126.7	124.4	125.8
∠ (C_α_–N_p_–C_α_)	107.7	111.8	108.8	111.7
∠ (N_t_–S–N_t_)		100.2		100.3

^1^ Bond lengths in Å and bond angles in degrees. ^2^ X is dummy atom located in center between N_p_ atoms.

**Table 2:** Selected parameters of MPz and MTTDPz complexes from NBO and quantum theory of atoms in molecules (QTAIM) calculations.

	CaPz	ZnPz	CaTTDPz	ZnTTDPz
*E*(HOMO),eV	−5.73	−5.99	−6.07	−6.19
*E*(LUMO),eV	−3.10	−3.33	−3.78	−3.91
∆*E*, eV	2.64	2.66	2.29	2.29
∇^2^ρ, a.u.	0.219	0.394	0.207	0.339
δ(M|N_p_)	0.270	0.464	0.262	0.446
*q*(M) NPA	1.754	1.198	1.768	1.234
*q*(N_p_) NPA	−0.702	−0.633	−0.660	−0.596
configuration	4s^0.12^3d^0.14^	4s^0.36^3d^9.96^4p^0.48^	4s^0.11^3d^0.13^	4s^0.35^3d^9.97^4p^0.44^
∑ E(d-a), kcal/mol	18	116	17	103
Q(M-N_p_)	0.110	0.336	0.104	0.321
r(M-N_p_)	2.276	1.979	2.299	2.025

**Table 3:** Calculated composition of the lowest excited states and corresponding oscillator strengths for MPz and MTTDPz complexes (M = Ca and Zn).

State	Composition (%)	λ, nm	f	exp λ, nm
**CaPz**				
1 ^1^E	$2a_{1}\to e^{*}$ (18) $a_{2}\to e^{*}$ (80)	513	0.16	
4 ^1^E	$1a_{1}\to e^{*}$ (33) $2a_{1}\to e^{*}$ (53) $a_{2}\to e^{*}$ (9)	344	0.21	
5 ^1^E	$1a_{1}\to e^{*}$ (62) $2a_{1}\to e^{*}$ (25) $a_{2}\to e^{*}$ (9)	308	0.59	
10 ^1^E	$e\to b_{1}^{*}$ (99)	238	0.06	
**CaTTDPz**				
1 ^1^E	$3a_{1}\to 1e^{*}$ (7) $3a_{2}\to 1e^{*}$ (90)	585	0.27	647 (Py) [this work] 641 (acetone) [this work]
6 ^1^E	$3a_{1}\to 1e^{*}$ (74) $3a_{2}\to 1e^{*}$ (8) $3a_{2}\to 2e^{*}$ (8)	322	0.98	
16 ^1^E	$1b_{1}\to 1e^{*}$ (9) $5e\to a_{1}^{*}$ (14) $3a_{1}\to 2e^{*}$ (67)	254	0.28	
17 ^1^E	$1b_{1}\to 1e^{*}$ (7) $2b_{2}\to 2e^{*}$ (77)	251	0.15	
18 ^1^E	$1a_{2}\to 1e^{*}$ (6) $1b_{1}\to 1e^{*}$ (30) $5e\to a_{1}^{*}$ (34) $5e\to b_{2}^{*}$ (5) $3a_{1}\to 2e^{*}$ (23)	250	0.14	
**ZnPz**				
1 ^1^E_u_	$2a_{2u}\to e_{g}^{*}$ (17) $a_{1u}\to e_{g}^{*}$ (82)	505	0.17	584 (Py) [27]
3 ^1^E_u_	$1a_{2u}\to e_{g}^{*}$ (50) $b_{2u}\to e_{g}^{*}$ (6) $2a_{2u}\to e_{g}^{*}$ (37) $a_{1u}\to e_{g}^{*}$ (6)	329	0.15	
4 ^1^E_u_	$1a_{2u}\to e_{g}^{*}$ (44) $2a_{2u}\to e_{g}^{*}$ (42) $a_{1u}\to e_{g}^{*}$ (11)	307	0.71	327
5 ^1^E_u_	$e_{g}\to b_{1u}^{*}$ (99)	238	0.06	
**ZnTTDPz**				
1 ^1^E_u_	$2a_{2u}\to 1e_{g}^{*}$ (5) $2a_{1u}\to 1e_{g}^{*}$ (91)	580	0.29	638 (DMSO) [23] 44 (DMF) [8]
4 ^1^E_u_	$1a_{2u}\to 1e_{g}^{*}$ (44) $2a_{2u}\to 1e_{g}^{*}$ (42) $b_{2u}\to 1e_{g}^{*}$ (11)	334	0.28	400
5 ^1^E_u_	$1a_{2u}\to 1e_{g}^{*}$ (39) $2a_{2u}\to 1e_{g}^{*}$ (42) $2a_{1u}\to 1e_{g}^{*}$ (7) $2a_{1u}\to 2e_{g}^{*}$ (6)	312	0.81	372
8 ^1^E_u_	$1a_{1u}\to 1e_{g}^{*}$ (6) $b_{1u}\to 1e_{g}^{*}$ (29) $2e_{g}\to b_{2u}^{*}$ (8) $2e_{g}\to a_{2u}^{*}$ (52)	252	0.55	320
9 ^1^E_u_	$2e_{g}\to a_{2u}^{*}$ (6) $b_{2u}\to 2e_{g}^{*}$ (86)	246	0.05	
12 ^1^E_u_	$1a_{1u}\to 1e_{g}^{*}$ (52) $1a_{2u}\to 2e_{g}^{*}$ (17) $2a_{2u}\to 2e_{g}^{*}$ (18) $b_{2u}\to 2e_{g}^{*}$ (6)	230	0.10	

## Supplementary Materials

Supplementary materials can be found at https://www.mdpi.com/1422-0067/21/8/2923/s1.

## Abbreviations

Pz	Porphyrazine
TTDPz	Tetrakis(1,2,5-thiadiazole) porphyrazine
DFT	Density Functional Theory
TDDFT	Time Dependent Density Functional Theory
NBO	Natural bond orbital
QTAIM	Quantum theory of atoms in molecules
