# Supplementary material for: DFT Study of Molecular and Electronic Structure of Ca(II) and Zn(II) Complexes with Porphyrazine and tetrakis(1,2,5-thiadiazole)porphyrazine
Source: Int J Mol Sci. 2020 Apr 22;21(8):2923. doi: 10.3390/ijms21082923 (PMC7215693; doi:10.3390/ijms21082923)
Supplement: Supplementary file 1 [file ijms-21-02923-s001.pdf]

Supporting information for

# «DFT Study of molecular and electronic structure of Ca(II) and Zn(II) complexes with porphyrazine and tetrakis(1,2,5-thiadiazole)porphyrazine»

Arseniy A. Otlyotov, Igor V. Ryzhov, Ilya A. Kuzmin, Yuriy A. Zhabanov, Maxim S. Mikhailov and Pavel A. Stuzhin

*Ivanovo State University of Chemistry and Technology, Research Institute of Chemistry of Macroheterocyclic Compounds, Sheremetievskiy av. 7, 153000 Ivanovo, Russian Federation*

## Contents:

Optimized Cartesian coordinates of CaPz optimized B3LYP/pcseg-2 level of theory: ........ 2

Optimized Cartesian coordinates of CaTTDPz optimized B3LYP/pcseg-2 level of theory: ........ 2

Optimized Cartesian coordinates of ZnPz optimized B3LYP/pcseg-2 level of theory: ........ 3

Optimized Cartesian coordinates of ZnTTDPz optimized B3LYP/pcseg-2 level of theory: ........ 4

Bond lengths and topological parameters of $\rho$(r) in bond critical points of the CaPz: ........ 5

Bond lengths and topological parameters of $\rho$(r) in bond critical points of the ZnPz: ........ 5

Bond lengths and topological parameters of $\rho$(r) in bond critical points of the CaTTDPz: ........ 5

Bond lengths and topological parameters of $\rho$(r) in bond critical points of the ZnTTDPz: ........ 6

Charge on atoms in CaPz, ZnPz, CaTTDPz and ZnTTDPz molecules: ........ 6

Electronic absorption spectra of the CaTTDPz in pyridine (a), in acetone (B). ........ 7

**Optimized Cartesian coordinates of CaPz optimized B3LYP/pcseg-2 level of theory:**

| | | | |
|---|---|---|---|
| N | -0.092626000000 | 2.001736000000 | 0.182671000000 |
| N | -2.001736000000 | -0.092626000000 | 0.182671000000 |
| N | 0.092626000000 | -2.001736000000 | 0.182671000000 |
| N | 2.001736000000 | 0.092626000000 | 0.182671000000 |
| N | 2.259202000000 | 2.478426000000 | -0.123037000000 |
| N | -2.478426000000 | 2.259202000000 | -0.123037000000 |
| N | -2.259202000000 | -2.478426000000 | -0.123037000000 |
| N | 2.478426000000 | -2.259202000000 | -0.123037000000 |
| C | 0.972044000000 | 2.817835000000 | -0.063189000000 |
| C | -2.817835000000 | 0.972044000000 | -0.063189000000 |
| C | -0.972044000000 | -2.817835000000 | -0.063189000000 |
| C | 2.716027000000 | 1.228113000000 | -0.063189000000 |
| C | -1.228113000000 | 2.716027000000 | -0.063189000000 |
| C | -2.716027000000 | -1.228113000000 | -0.063189000000 |
| C | 1.228113000000 | -2.716027000000 | -0.063189000000 |
| C | 2.817835000000 | -0.972044000000 | -0.063189000000 |
| C | 0.485706000000 | 4.153000000000 | -0.391589000000 |
| C | -4.153000000000 | 0.485706000000 | -0.391589000000 |
| C | -0.485706000000 | -4.153000000000 | -0.391589000000 |
| C | 4.090399000000 | 0.867152000000 | -0.391589000000 |
| C | -0.867152000000 | 4.090399000000 | -0.391589000000 |
| C | -4.090399000000 | -0.867152000000 | -0.391589000000 |
| C | 0.867152000000 | -4.090399000000 | -0.391589000000 |
| C | 4.153000000000 | -0.485706000000 | -0.391589000000 |
| H | 1.123852000000 | 4.985879000000 | -0.634781000000 |
| H | -4.985879000000 | 1.123852000000 | -0.634781000000 |
| H | -1.123852000000 | -4.985879000000 | -0.634781000000 |
| H | 4.860788000000 | 1.579486000000 | -0.634781000000 |
| H | -1.579486000000 | 4.860788000000 | -0.634781000000 |
| H | 4.860788000000 | -1.579486000000 | -0.634781000000 |
| H | 1.579486000000 | -4.860788000000 | -0.634781000000 |
| H | 4.985879000000 | -1.123852000000 | -0.634781000000 |
| Ca | 0.000000000000 | 0.000000000000 | 1.261894000000 |

**Optimized Cartesian coordinates of CaTTDPz optimized B3LYP/pcseg-2 level of theory:**

| | | | |
|---|---|---|---|
| N | -0.974056000000 | 1.815121000000 | 0.364154000000 |
| N | -1.815121000000 | -0.974056000000 | 0.364154000000 |
| N | 0.974056000000 | -1.815121000000 | 0.364154000000 |
| N | 1.815121000000 | 0.974056000000 | 0.364154000000 |
| N | -3.236805000000 | 0.976045000000 | 0.143599000000 |
| N | -0.976045000000 | -3.236805000000 | 0.143599000000 |
| N | 3.236805000000 | -0.976045000000 | 0.143599000000 |
| N | 0.976045000000 | 3.236805000000 | 0.143599000000 |
| C | -2.329072000000 | 1.936326000000 | 0.181263000000 |
| C | -1.936326000000 | -2.329072000000 | 0.181263000000 |
| C | 2.329072000000 | -1.936326000000 | 0.181263000000 |
| C | 3.011259000000 | 0.325973000000 | 0.181263000000 |
| C | -0.325973000000 | 3.011259000000 | 0.181263000000 |
| C | -3.011259000000 | -0.325973000000 | 0.181263000000 |
| C | 0.325973000000 | -3.011259000000 | 0.181263000000 |
| C | 1.936326000000 | 2.329072000000 | 0.181263000000 |
| C | -2.606418000000 | 3.351210000000 | -0.060310000000 |
| C | -3.351210000000 | -2.606418000000 | -0.060310000000 |
| C | 2.606418000000 | -3.351210000000 | -0.060310000000 |
| C | 4.024552000000 | 1.351670000000 | -0.060310000000 |

| | | | |
|---|---|---|---|
| C | -1.351670000000 | 4.024552000000 | -0.060310000000 |
| C | -4.024552000000 | -1.351670000000 | -0.060310000000 |
| C | 1.351670000000 | -4.024552000000 | -0.060310000000 |
| C | 3.351210000000 | 2.606418000000 | -0.060310000000 |
| N | -3.645851000000 | 4.123839000000 | -0.291749000000 |
| N | -4.123839000000 | -3.645851000000 | -0.291749000000 |
| N | 3.645851000000 | -4.123839000000 | -0.291749000000 |
| N | 5.317836000000 | 1.420879000000 | -0.291749000000 |
| N | -1.420879000000 | 5.317836000000 | -0.291749000000 |
| N | -5.317836000000 | -1.420879000000 | -0.291749000000 |
| N | 1.420879000000 | -5.317836000000 | -0.291749000000 |
| N | 4.123839000000 | 3.645851000000 | -0.291749000000 |
| S | -3.023204000000 | 5.633637000000 | -0.490092000000 |
| S | -5.633637000000 | -3.023204000000 | -0.490092000000 |
| S | 3.023204000000 | -5.633637000000 | -0.490092000000 |
| S | 5.633637000000 | 3.023204000000 | -0.490092000000 |
| Ca | 0.000000000000 | 0.000000000000 | 1.384053000000 |

**Optimized Cartesian coordinates of ZnPz optimized B3LYP/pcseg-2 level of theory:**

| | | | |
|---|---|---|---|
| N | 0.000000000000 | 1.978859000000 | 0.000000000000 |
| N | -1.978859000000 | 0.000000000000 | 0.000000000000 |
| N | 0.000000000000 | -1.978859000000 | 0.000000000000 |
| N | 1.978859000000 | 0.000000000000 | 0.000000000000 |
| N | 2.379067000000 | 2.379067000000 | 0.000000000000 |
| N | -2.379067000000 | 2.379067000000 | 0.000000000000 |
| N | -2.379067000000 | -2.379067000000 | 0.000000000000 |
| N | 2.379067000000 | -2.379067000000 | 0.000000000000 |
| C | 1.108009000000 | 2.772569000000 | 0.000000000000 |
| C | -2.772569000000 | 1.108009000000 | 0.000000000000 |
| C | -1.108009000000 | -2.772569000000 | 0.000000000000 |
| C | 2.772569000000 | 1.108009000000 | 0.000000000000 |
| C | -1.108009000000 | 2.772569000000 | 0.000000000000 |
| C | -2.772569000000 | -1.108009000000 | 0.000000000000 |
| C | 1.108009000000 | -2.772569000000 | 0.000000000000 |
| C | 2.772569000000 | -1.108009000000 | 0.000000000000 |
| C | 0.677767000000 | 4.164526000000 | 0.000000000000 |
| C | -4.164526000000 | 0.677767000000 | 0.000000000000 |
| C | -0.677767000000 | -4.164526000000 | 0.000000000000 |
| C | 4.164526000000 | 0.677767000000 | 0.000000000000 |
| C | -0.677767000000 | 4.164526000000 | 0.000000000000 |
| C | -4.164526000000 | -0.677767000000 | 0.000000000000 |
| C | 0.677767000000 | -4.164526000000 | 0.000000000000 |
| C | 4.164526000000 | -0.677767000000 | 0.000000000000 |
| H | 1.351226000000 | 5.004849000000 | 0.000000000000 |
| H | -5.004849000000 | 1.351226000000 | 0.000000000000 |
| H | -1.351226000000 | -5.004849000000 | 0.000000000000 |
| H | 5.004849000000 | 1.351226000000 | 0.000000000000 |
| H | -1.351226000000 | 5.004849000000 | 0.000000000000 |
| H | -5.004849000000 | -1.351226000000 | 0.000000000000 |
| H | 1.351226000000 | -5.004849000000 | 0.000000000000 |
| H | 5.004849000000 | -1.351226000000 | 0.000000000000 |
| Zn | 0.000000000000 | 0.000000000000 | 0.000000000000 |

**Optimized Cartesian coordinates of ZnTTDPz optimized B3LYP/pcseg-2 level of theory:**

| | | | |
|---|---|---|---|
| N | 0.000000000000 | 2.024496000000 | 0.000000000000 |
| N | -2.024496000000 | 0.000000000000 | 0.000000000000 |
| N | 0.000000000000 | -2.024496000000 | 0.000000000000 |
| N | 2.024496000000 | 0.000000000000 | 0.000000000000 |
| N | -2.391263000000 | 2.391263000000 | 0.000000000000 |
| N | -2.391263000000 | -2.391263000000 | 0.000000000000 |
| N | 2.391263000000 | -2.391263000000 | 0.000000000000 |
| N | 2.391263000000 | 2.391263000000 | 0.000000000000 |
| C | -1.137996000000 | 2.796116000000 | 0.000000000000 |
| C | -2.796116000000 | -1.137996000000 | 0.000000000000 |
| C | 1.137996000000 | -2.796116000000 | 0.000000000000 |
| C | 2.796116000000 | -1.137996000000 | 0.000000000000 |
| C | 1.137996000000 | 2.796116000000 | 0.000000000000 |
| C | -2.796116000000 | 1.137996000000 | 0.000000000000 |
| C | -1.137996000000 | -2.796116000000 | 0.000000000000 |
| C | 2.796116000000 | 1.137996000000 | 0.000000000000 |
| C | -0.710604000000 | 4.190394000000 | 0.000000000000 |
| C | -4.190394000000 | -0.710604000000 | 0.000000000000 |
| C | 0.710604000000 | -4.190394000000 | 0.000000000000 |
| C | 4.190394000000 | -0.710604000000 | 0.000000000000 |
| C | 0.710604000000 | 4.190394000000 | 0.000000000000 |
| C | -4.190394000000 | 0.710604000000 | 0.000000000000 |
| C | -0.710604000000 | -4.190394000000 | 0.000000000000 |
| C | 4.190394000000 | 0.710604000000 | 0.000000000000 |
| N | -1.262118000000 | 5.385704000000 | 0.000000000000 |
| N | -5.385704000000 | -1.262118000000 | 0.000000000000 |
| N | 1.262118000000 | -5.385704000000 | 0.000000000000 |
| N | 5.385704000000 | -1.262118000000 | 0.000000000000 |
| N | 1.262118000000 | 5.385704000000 | 0.000000000000 |
| N | -5.385704000000 | 1.262118000000 | 0.000000000000 |
| N | -1.262118000000 | -5.385704000000 | 0.000000000000 |
| N | 5.385704000000 | 1.262118000000 | 0.000000000000 |
| S | 0.000000000000 | 6.438480000000 | 0.000000000000 |
| S | -6.438480000000 | 0.000000000000 | 0.000000000000 |
| S | 0.000000000000 | -6.438480000000 | 0.000000000000 |
| S | 6.438480000000 | 0.000000000000 | 0.000000000000 |
| Zn | 0.000000000000 | 0.000000000000 | 0.000000000000 |

**Bond lengths and topological parameters of $\rho$(r) in bond critical points of the CaPz:**

| interaction | $r_e$ (Å) | $\rho$ (a.u.) | $\nabla^2\rho$ (a.u.) | $\lambda_1$ | $\lambda_2$ | $\lambda_3$ | ε | $G_b$(a.u.) | $V_b$(a.u.) | $H_b$(a.u.) | δ(A\|B) |
|---|---|---|---|---|---|---|---|---|---|---|---|
| **$N_m$-$C_{\alpha1,2}$** | 1,332 | 0,354 | -1,245 | -0,856 | -0,743 | 0,354 | 0,152 | 0,222 | -0,756 | 0,534 | 1,240 |
| **$N_p$-$C_{\alpha1,2}$** | 1,364 | 0,336 | -1,085 | -0,789 | -0,695 | 0,398 | 0,135 | 0,178 | -0,627 | 0,449 | 1,156 |
| **$N_p$–Ca** | 2,276 | 0,054 | 0,219 | -0,075 | -0,066 | 0,360 | 0,139 | 0,055 | -0,055 | 0,000 | 0,270 |
| **$C_{\alpha1,2}$-$C_{\beta1,2}$** | 1,458 | 0,286 | -0,759 | -0,612 | -0,538 | 0,391 | 0,139 | 0,078 | -0,346 | 0,268 | 1,061 |
| **$C_{\beta1,2}$-H** | 1,077 | 0,296 | -1,215 | -0,832 | -0,821 | 0,437 | 0,014 | 0,042 | -0,388 | 0,346 | 0,967 |
| **$C_{\beta1}$-$C_{\beta2}$** | 1,354 | 0,341 | -0,990 | -0,765 | -0,609 | 0,384 | 0,256 | 0,130 | -0,508 | 0,378 | 1,604 |

**Bond lengths and topological parameters of $\rho$(r) in bond critical points of the ZnPz:**

| interaction | $r_e$ (Å) | $\rho$ (a,u,) | $\nabla^2\rho$ (a,u,) | $\lambda_1$ | $\lambda_2$ | $\lambda_3$ | ε | $G_b$(a,u,) | $V_b$(a,u,) | $H_b$(a,u,) | δ(A\|B) |
|---|---|---|---|---|---|---|---|---|---|---|---|
| **$N_m$-$C_{\alpha1,2}$** | 1,331 | 0,356 | -1,258 | -0,863 | -0,748 | 0,353 | 0,153 | 0,225 | -0,764 | 0,539 | 1,243 |
| **$N_p$-$C_{\alpha1,2}$** | 1,363 | 0,336 | -1,100 | -0,792 | -0,696 | 0,387 | 0,138 | 0,184 | -0,642 | 0,459 | 1,130 |
| **$N_p$–Zn** | 1,979 | 0,094 | 0,394 | -0,131 | -0,124 | 0,649 | 0,061 | 0,122 | -0,146 | 0,024 | 0,464 |
| **$C_{\alpha1,2}$-$C_{\beta1,2}$** | 1,457 | 0,286 | -0,764 | -0,615 | -0,540 | 0,391 | 0,138 | 0,079 | -0,348 | 0,270 | 1,064 |
| **$C_{\beta1,2}$-H** | 1,077 | 0,296 | -1,222 | -0,835 | -0,824 | 0,437 | 0,013 | 0,041 | -0,388 | 0,347 | 0,966 |
| **$C_{\beta1}$-$C_{\beta2}$** | 1,355 | 0,340 | -0,986 | -0,763 | -0,607 | 0,385 | 0,256 | 0,130 | -0,506 | 0,376 | 1,603 |

**Bond lengths and topological parameters of $\rho$(r) in bond critical points of the CaTTDPz:**

| interaction | $r_e$ (Å) | $\rho$ (a,u,) | $\nabla^2\rho$ (a,u,) | $\lambda_1$ | $\lambda_2$ | $\lambda_3$ | ε | $G_b$(a,u,) | $V_b$(a,u,) | $H_b$(a,u,) | δ(A\|B) |
|---|---|---|---|---|---|---|---|---|---|---|---|
| **$N_m$-$C_{\alpha1,2}$** | 1,322 | 0,360 | -1,272 | -0,875 | -0,747 | 0,350 | 0,172 | 0,239 | -0,796 | 0,557 | 1,256 |
| **$N_p$-$C_{\alpha1,2}$** | 1,373 | 0,328 | -1,030 | -0,765 | -0,665 | 0,400 | 0,150 | 0,175 | -0,608 | 0,433 | 1,153 |
| **$N_p$–Ca** | 2,299 | 0,052 | 0,207 | -0,071 | -0,062 | 0,340 | 0,135 | 0,052 | -0,052 | 0,000 | 0,262 |
| **$C_{\alpha1,2}$-$C_{\beta1,2}$** | 1,462 | 0,283 | -0,756 | -0,611 | -0,535 | 0,390 | 0,143 | 0,075 | -0,339 | 0,264 | 1,002 |
| **$C_{\beta1,2}$–$N_{25-32}$** | 1,316 | 0,361 | -1,172 | -0,841 | -0,724 | 0,393 | 0,161 | 0,299 | -0,891 | 0,592 | 1,355 |
| **$C_{\beta1}$-$C_{\beta2}$** | 1,424 | 0,310 | -0,860 | -0,687 | -0,586 | 0,414 | 0,172 | 0,095 | -0,405 | 0,310 | 1,089 |
| **$N_{25-32}$–$S_{33-36}$** | 1,645 | 0,234 | -0,347 | -0,373 | -0,275 | 0,301 | 0,354 | 0,176 | -0,438 | 0,263 | 1,304 |

**Bond lengths and topological parameters of $\rho$(r) in bond critical points of the ZnTTDPz:**

| interaction | $r_e$ (Å) | $\rho$ (a.u.) | $\nabla^2\rho$ (a.u.) | $\lambda_1$ | $\lambda_2$ | $\lambda_3$ | ε | $G_b$(a.u.) | $V_b$(a.u.) | $H_b$(a.u.) | δ(A\|B) |
|---|---|---|---|---|---|---|---|---|---|---|---|
| **$N_m$-$C_{\alpha1,2}$** | 1,317 | 0,365 | -1,303 | -0,893 | -0,761 | 0,350 | 0,173 | 0,247 | -0,819 | 0,573 | 1,258 |
| **$N_p$-$C_{\alpha1,2}$** | 1,375 | 0,326 | -1,025 | -0,760 | -0,659 | 0,394 | 0,155 | 0,177 | -0,610 | 0,433 | 1,127 |
| **$N_p$–Zn** | 2,024 | 0,085 | 0,339 | -0,116 | -0,109 | 0,564 | 0,059 | 0,105 | -0,124 | 0,020 | 0,446 |
| **$C_{\alpha1,2}$-$C_{\beta1,2}$** | 1,458 | 0,285 | -0,768 | -0,618 | -0,540 | 0,390 | 0,144 | 0,076 | -0,343 | 0,268 | 1,006 |
| **$C_{\beta1,2}$–$N_{25\text{-}32}$** | 1,316 | 0,361 | -1,173 | -0,839 | -0,723 | 0,389 | 0,161 | 0,296 | -0,885 | 0,589 | 1,352 |
| **$C_{\beta1}$-$C_{\beta2}$** | 1,421 | 0,312 | -0,868 | -0,692 | -0,590 | 0,414 | 0,173 | 0,096 | -0,409 | 0,313 | 1,093 |
| **$N_{25\text{-}32}$–$S_{33\text{-}36}$** | 1,643 | 0,365 | -1,303 | -0,893 | -0,761 | 0,350 | 0,173 | 0,247 | -0,819 | 0,573 | 1,258 |

**Charge on atoms in CaPz, ZnPz, CaTTDPz and ZnTTDPz molecules:**

| Name | $N_{1\text{-}4}$ | $N_{5\text{-}8}$ | $C_{17\text{-}24}$ | $H_{25\text{-}32}$ | $C_{9\text{--}16}$ | $Ca_{33}$ | $Zn_{33}$ |
|---|---|---|---|---|---|---|---|
| q(A) CaPz | -1,169 | -1,127 | +0,003 | +0,025 | +0,924 | +1,564 | - |
| q(A) ZnPz | -1,160 | -1,119 | 0,010 | 0,032 | 0,940 | - | 1,255 |

| | $N_{1\text{-}4}$ | $N_{25\text{-}32}$ | $N_{5\text{-}8}$ | $C_{17\text{-}24}$ | $C_{9\text{--}16}$ | $S_{33\text{-}36}$ | $Ca_{37}$ | $Zn_{37}$ |
|---|---|---|---|---|---|---|---|---|
| q(A) CaTTDPz | -1,143 | -1,142 | -1,127 | +0,521 | +0,960 | +1,197 | +1,580 | - |
| q(A) ZnTTDPz | -1,123 | -1,138 | -1,128 | 0,523 | 0,975 | 1,215 | - | 1,258 |

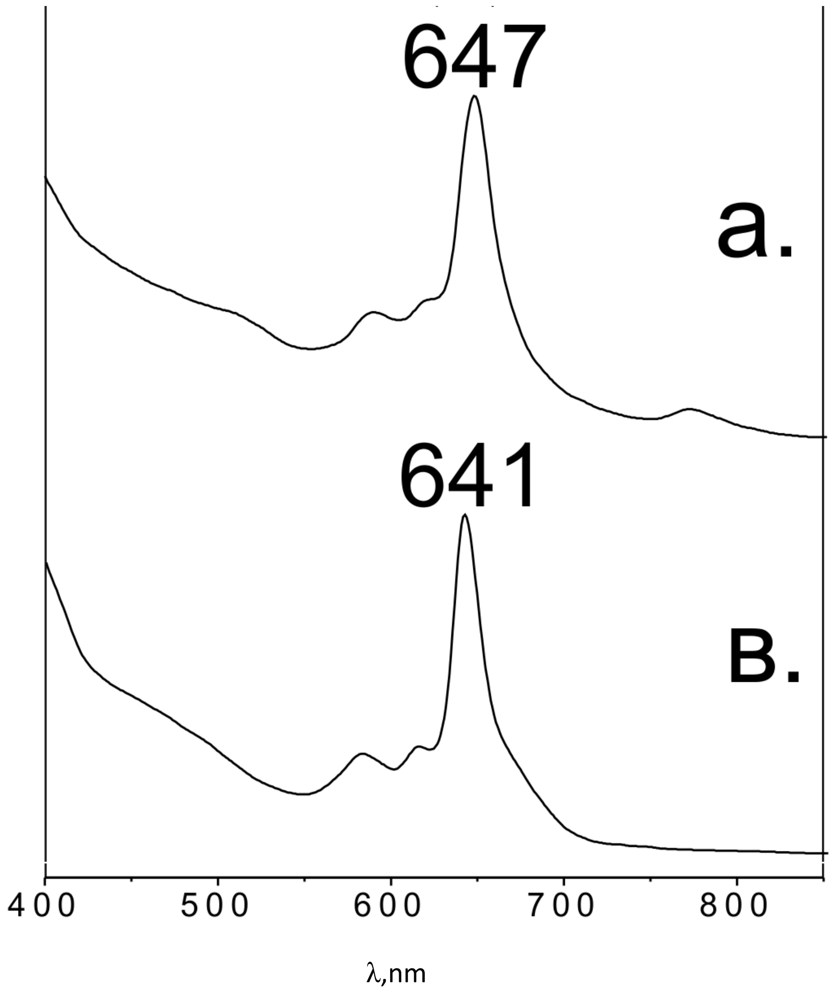


**Electronic absorption spectra of the CaTTDPz in pyridine (a), in acetone (B).**
